# Supplementary material for: Transcriptional Profiling of Cardiac Cells Links Age-Dependent Changes in Acetyl-CoA Signaling to Chromatin Modifications
Source: Int J Mol Sci. 2021 Jun 29;22(13):6987. doi: 10.3390/ijms22136987 (PMC8268808; doi:10.3390/ijms22136987)
Supplement: Supplementary file 1 [file ijms-22-06987-s001.zip › ijms-1267971 suppl/ijms-1267971 SUPPL.pdf]

Supplemental Materials and Methods

Cell Isolation and Culture
All cardiac tissue-derived stem like cells (CTSCs) were isolated from the heart tissue of C57BL/6 Mice (Jackson). The heart was digested, centrifuged and pellet was formed followed by plating in growth media growth media that includes DMEM-F12 (Gibco), 10% Embryonic Stem Cell Fetal Bovine Serum (Gibco), 1% Penicillin-Streptomycin-Glutamine (PSG) (Gibco), 1x Insulin-Transferrin-Selenium (Gibco), Recombinant Human-EGF 10ng/mL (Peprotech), Recombinant Human-FGF 10ng/mL (Peprotech), and Leukemia Inhibitory Factor 50,000 units (Millipore Sigma), and incubated in a 37°C 5% CO_2 incubator. Routine cell culturing passaging include 1x PBS wash (Gibco), dissociation of cells using 0.25% Trypsin EDTA (Gibco), centrifugation for 5 minutes at 1500RPM was carried out for the next 14 days that led to the emergence of CTSCs. Pelleted cells were resuspended in CTSCs Growth media and counted using a hemocytometer. Afterwards, cells were plated onto polystyrene tissue culture plates (Falcon Corning).

Immunoblot
Immunoblot analysis was done for CTSCs as described previously [1-3,6]. Briefly, 100uL of Protein Lysis buffer that consists of Na3VO4, NaF, 4% SDS/PBS, and Protease Inhibitor was added to the cells. Samples were then added to QIAShredder (Qiagen) and spun at max rpm for 10min. Flow-through sample was collected and protein quantification was performed using Pierce BCA Protein Assay Kit (Invitrogen). Samples were normalized to 20ug/well and western blot assay was performed. Protein samples were loaded onto 4-20% Mini-PROTEAN TGX Precast Protein Gels (Bio-Rad) for electrophoresis. After transfer, blot was washed in 1x TBS and blocked with Odyssey PBS Blocking Buffer (LI-COR) for 1 hour. Primary antibody incubation was performed overnight and washed in 1x TBS. Secondary antibody incubation was done for 90 minutes and washed with 0.1% TBST two times followed by 0.4% TBST. Antibody scanning was conducted using Odyssey CLx Western Blot Detection System (LI-COR). Antibody and dilutions are listed on Supplemental Table I.

Table S1 – List of primers

Primer Name	Forward/Reverse	Product size	Sequence
ACSS2	Forward	102	GCC ATA TGC TGA CCC CTC TC
ACSS2	Reverse		TCC CCG GAC TCA TTC AGG AT
ACLY	Forward	118	CTA TGC CCC AAG GAA AGA GTG
ACLY	Reverse		TCT CGG GAA CAC ACG TAG TC
CTP1a	Forward	118	GGACTCCGCTCGCTCATT
CTP1a	Reverse		GAGATCGATGCCATCAGGGG
PDHB	Forward	90	AGT TGC CCA GTA TGA CGG TG
PDHB	Reverse		TCT GAG ATG GGG GTG TCG AT
SLC25a1	Forward	76	GGAGGCACACAAATACCGGA
SLC25a1	Reverse		GTAGAATGCCTTTGGCCCCT

Table S2 – List of antibodies

Application	Antibody	Dilution	Cat No.	Company
Western Blot	Histone H3	1:100	9715	Cell Signaling
Western Blot	Acetyl Histone H3	1:100	06-599	Millipore
Western Blot	AceCS1	1:100	3658S	Cell Signaling
Western Blot	ATP Citrate Lyase	1:100	4332S	Cell Signaling
Western Blot	βactin	1:500	9664S	Cell Signaling

Supplementary Figure 1

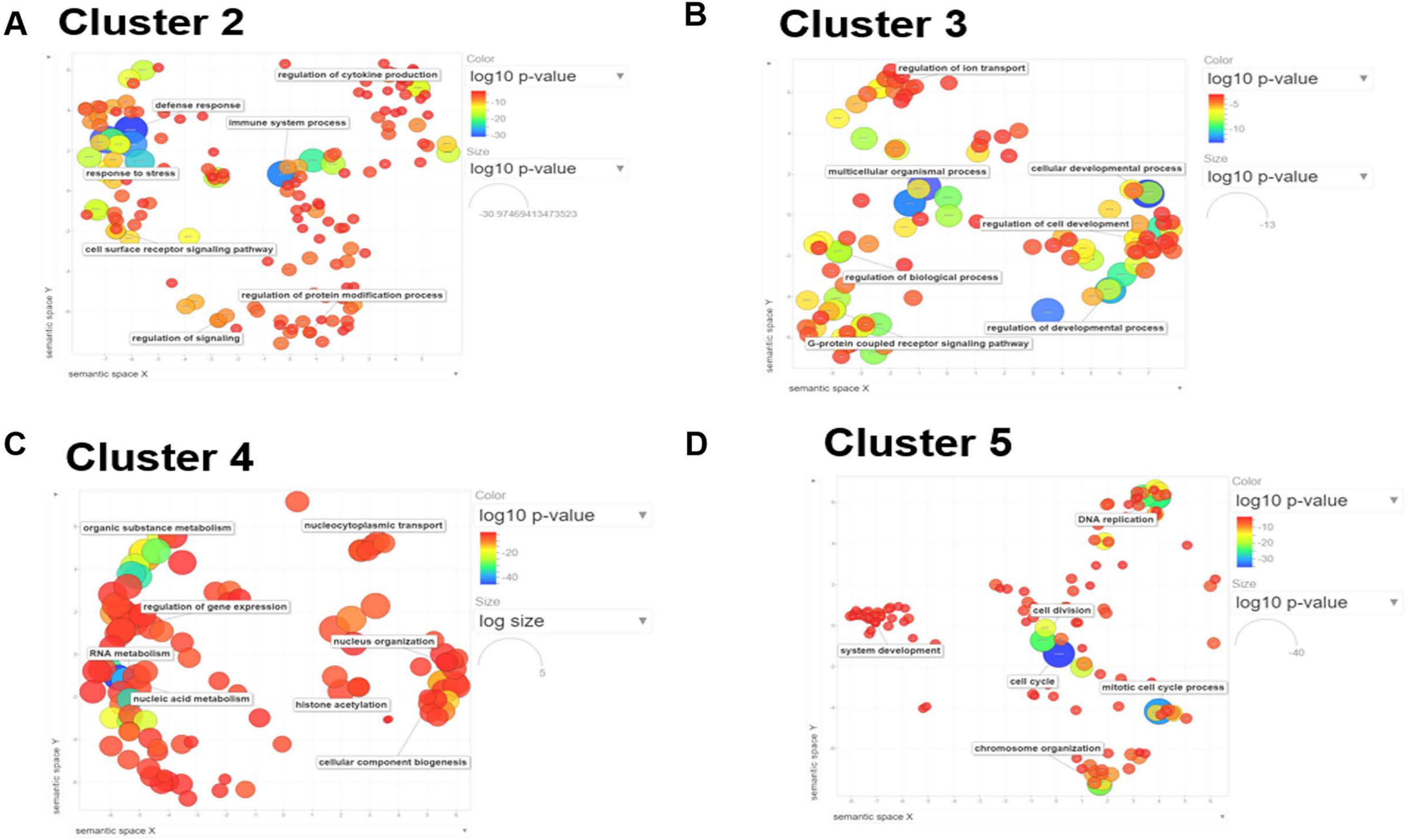

Supplementary Figure 2

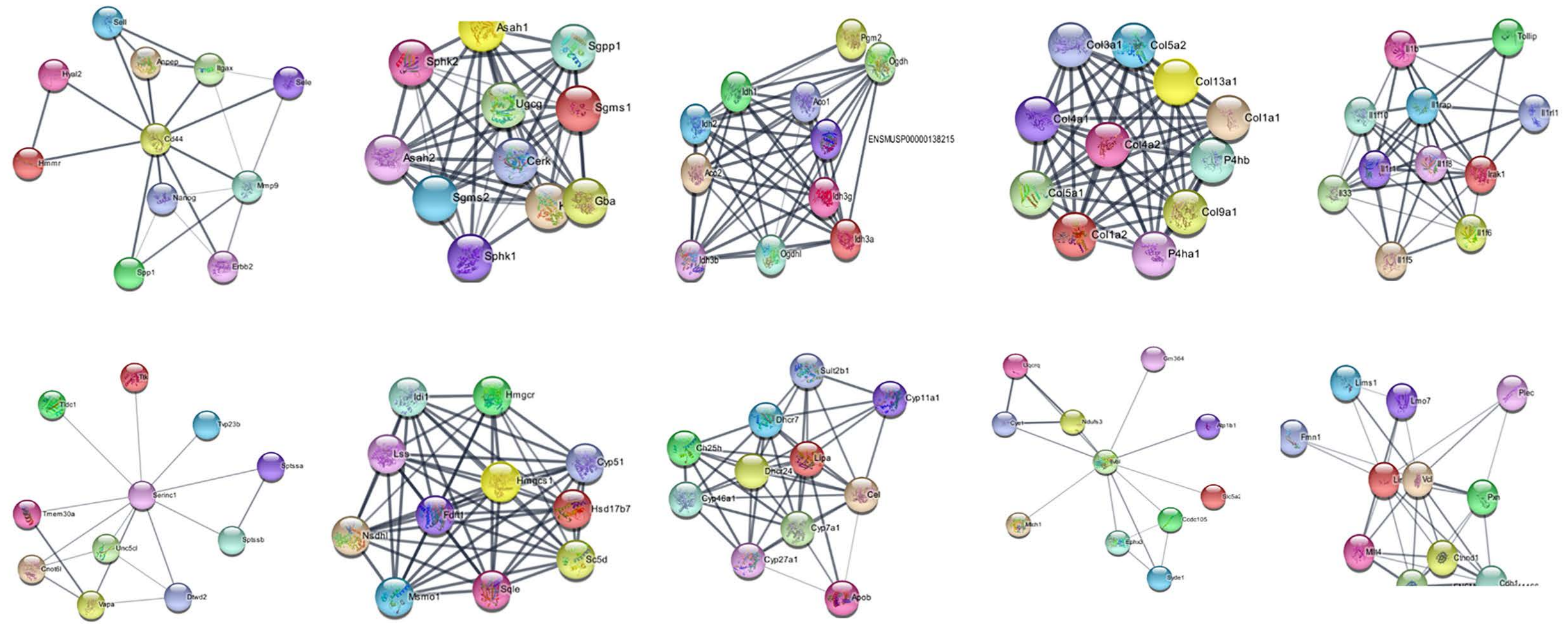

Supplementary Figure 3

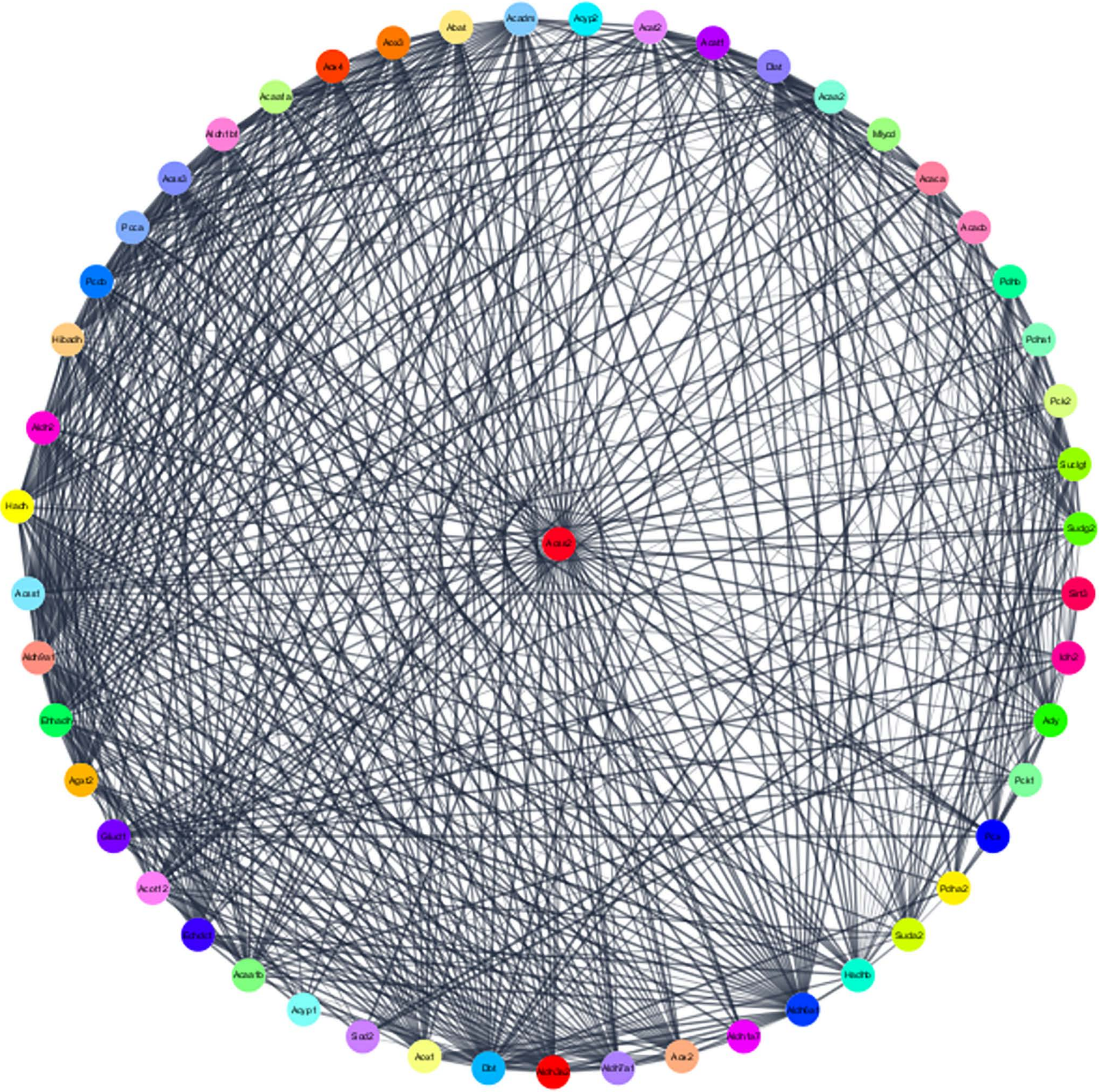

Top Gene	logFC	adj.p-value
**Lipa**	1.880428797	8.3778E-265
**Lima1**	1.386559217	4.9641E-287
**P4ha1**	1.216435527	4.0584E-292
**Idh1**	1.584898068	1.4915E-241
**Asah1**	0.963340936	1.8342E-167
**Il1rap**	2.253245162	4.1379E-303
**Serinc1**	0.986106666	7.161E-242
**Ilvbl**	2.185676922	1.048E-151
**Sqle**	0.920963676	4.2693E-134
**Cd44**	0.996198493	5.32E-285

	**Predicted Gene**	**Score**		
	Apob	0.926		
	Dhcr7	0.924		
	Dhcr24	0.905		
	Cel	0.889		
**Lipa**	Ch25h	0.869		**Lima1**
	Cyp27a1	0.861		
	Cyp7a1	0.853		
	Cyp11a1	0.814		
	Cyp46a1	0.813		
	Sult2b1	0.804		
	**Predicted Gene**	**Score**		
	Il33	0.994		
	Il1b	0.992		
	Il1rl1	0.992		
	Il1r1	0.986		
**Il1rap**	Il1f5	0.979		**Serinc1**
	Irak1	0.979		
	Il1f8	0.978		
	Il1f6	0.975		
	Tollip	0.97		
	Il1f10	0.968		

Predicted Gene	Score			Predicted Gene	Score	
Cdh1	0.727			P4hb	0.966	
Vcl	0.721			Col1a1	0.947	
Pxn	0.648			Col3a1	0.939	
Ctnnd1	0.6			Col13a1	0.938	
ENSMUSG00000101645	0.593		**P4ha1**	Col5a1	0.936	
Lmo7	0.58			Col1a2	0.932	
Fmn1	0.543			Col4a1	0.929	
Mllt4	0.54			Col4a2	0.928	
Lims1	0.514			Col9a1	0.924	
Plec	0.508			Col5a2	0.924	
**Predicted Gene**	**Score**			**Predicted Gene**	**Score**	
Vapa	0.745			Syde1	0.705	
Dtwd2	0.674			Cyc1	0.7	
Sptssa	0.613			Ndufs3	0.695	
Cnot6l	0.612			Atp1b1	0.691	
Tvp23b	0.591		**Ilvbl**	Uqcrq	0.668	
Sptssb	0.589			Ephx3	0.62	
Tmem30a	0.566			Gm364	0.573	
Tldc1	0.524			Ccdc105	0.536	
Ttk	0.523			Slc5a2	0.532	
Unc5cl	0.514			Mtch1	0.531	

	**Predicted Gene**	**Score**			**Predicted Gene**	**Score**
	Idh3g	0.987			Cerk	0.969
	Idh3a	0.987			Ugcg	0.968
	Idh3b	0.985			Sphk2	0.964
	Ogdh	0.972			Sphk1	0.964
**Idh1**	Aco2	0.972		**Asah1**	Asah2	0.962
	ENSMUSG0000008423	0.965			Kdsr	0.959
	Aco1	0.964			Gba	0.958
	Pgm2	0.953			Sgms2	0.955
	Idh2	0.951			Sgms1	0.953
	Ogdhl	0.946			Sgpp1	0.951
	**Predicted Gene**	**Score**			**Predicted Gene**	**Score**
	Lss	0.998			Sell	0.991
	Cyp51	0.996			Spp1	0.99
	Msmo1	0.996			Sele	0.987
	Fdft1	0.996			Hmmr	0.985
**Sqle**	Idi1	0.992		**Cd44**	Mmp9	0.982
	Hmgcs1	0.991			Itgax	0.981
	Hmgcr	0.985			Hyal2	0.979
	Nsdhl	0.983			Anpep	0.976
	Hsd17b7	0.958			Nanog	0.97
	Sc5d	0.958			Erbb2	0.969
