# Supplementary material for: Transcriptional Profiling of Cardiac Cells Links Age-Dependent Changes in Acetyl-CoA Signaling to Chromatin Modifications
Source: Int J Mol Sci. 2021 Jun 29;22(13):6987. doi: 10.3390/ijms22136987 (PMC8268808; doi:10.3390/ijms22136987)
Supplement: Supplementary file 1 [file ijms-22-06987-s001.zip › ijms-1267971 suppl/Table S3 - Top 10 genes in Cluster 1 networks.pdf]

Table S3: Top 10 genes in Cluster 1 networks

Top Gene	logFC	adj.p-value
**Lipa**	1.880428797	8.3778E-265
**Lima1**	1.386559217	4.9641E-287
**P4ha1**	1.216435527	4.0584E-292
**Idh1**	1.584898068	1.4915E-241
**Asah1**	0.963340936	1.8342E-167
**Il1rap**	2.253245162	4.1379E-303
**Serinc1**	0.986106666	7.161E-242
**Ilvbl**	2.185676922	1.048E-151
**Sqle**	0.920963676	4.2693E-134
**Cd44**	0.996198493	5.32E-285

**Lipa**	**Predicted Gene**	**Score**		**Lima1**
	Apob	0.926		
	Dhcr7	0.924		
	Dhcr24	0.905		
	Cel	0.889		
	Ch25h	0.869		
	Cyp27a1	0.861		
	Cyp7a1	0.853		
	Cyp11a1	0.814		
	Cyp46a1	0.813		
	Sult2b1	0.804		
**Il1rap**	**Predicted Gene**	**Score**		**Serinc1**
	Il33	0.994		
	Il1b	0.992		
	Il1rl1	0.992		
	Il1r1	0.986		
	Il1f5	0.979		
	Irak1	0.979		
	Il1f8	0.978		
	Il1f6	0.975		
	Tollip	0.97		
	Il1f10	0.968		

Predicted Gene	Score			Predicted Gene	Score	
Cdh1	0.727			P4hb	0.966	
Vcl	0.721			Col1a1	0.947	
Pxn	0.648			Col3a1	0.939	
Ctnnd1	0.6			Col13a1	0.938	
ENSMUSG00000101645	0.593		**P4ha1**	Col5a1	0.936	
Lmo7	0.58			Col1a2	0.932	
Fmn1	0.543			Col4a1	0.929	
Mllt4	0.54			Col4a2	0.928	
Lims1	0.514			Col9a1	0.924	
Plec	0.508			Col5a2	0.924	
**Predicted Gene**	**Score**			**Predicted Gene**	**Score**	
Vapa	0.745			Syde1	0.705	
Dtwd2	0.674			Cyc1	0.7	
Sptssa	0.613			Ndufs3	0.695	
Cnot6l	0.612			Atp1b1	0.691	
Tvp23b	0.591		**Ilvbl**	Uqcrq	0.668	
Sptssb	0.589			Ephx3	0.62	
Tmem30a	0.566			Gm364	0.573	
Tldc1	0.524			Ccdc105	0.536	
Ttk	0.523			Slc5a2	0.532	
Unc5cl	0.514			Mtch1	0.531	

	Predicted Gene	Score			Predicted Gene	Score
	Idh3g	0.987			Cerk	0.969
	Idh3a	0.987			Ugcg	0.968
	Idh3b	0.985			Sphk2	0.964
	Ogdh	0.972			Sphk1	0.964
**Idh1**	Aco2	0.972		**Asah1**	Asah2	0.962
	ENSMUSG0000008423	0.965			Kdsr	0.959
	Aco1	0.964			Gba	0.958
	Pgm2	0.953			Sgms2	0.955
	Idh2	0.951			Sgms1	0.953
	Ogdhl	0.946			Sgpp1	0.951
	**Predicted Gene**	**Score**			**Predicted Gene**	**Score**
	Lss	0.998			Sell	0.991
	Cyp51	0.996			Spp1	0.99
	Msmo1	0.996			Sele	0.987
	Fdft1	0.996			Hmmr	0.985
**Sqle**	Idi1	0.992		**Cd44**	Mmp9	0.982
	Hmgcs1	0.991			Itgax	0.981
	Hmgcr	0.985			Hyal2	0.979
	Nsdhl	0.983			Anpep	0.976
	Hsd17b7	0.958			Nanog	0.97
	Sc5d	0.958			Erbb2	0.969
